# Correspondence

**Published:** 1868-07-01

**Authors:** 


### CORRESPONDENCE.

"There is nothing new under the sun." — SOLOMON.
"We travel in circles." — GEN. SHERMAN.

MORE particularly were these axioms of wise and great men brought to my mind, by *two very recent discoveries*, and their truth demonstrated.

Very recently, in the western part of New York, is the claim for reduction of dislocated femur, by tact, without pullies or traction — or, in other words, make the muscles restore the integrity of the joint, without force or suffering — made by a surgeon; and, *mirabile dictu*, one and another claimed the discovery, each giving dates and facts to show precedence and originality. This is *singular*, and can only be accounted for by the axioms above.

In 1832, I graduated at the University of Maryland, in Baltimore city, and wrote my thesis "Upon the American Method of Reducing Dislocated Femurs." Prof. N. R. Smith (and may his shadow never grow less!) lectured, as he does now, on surgery, and gave us the history of cases reduced by his father, Prof. Nathan Smith, of Yale; exhibted drawings on the subject, and demonstrated the success of the plan. In compliment to the father, from love of the son, I wrote a thesis; had all of Cooper's plates of pullies, and other tortures, attached, and demonstrated, by plates and drawings, the feasibility and success of the operation; and gave the credit of discovery to the elder Smith. I shall never forget standing in the presence of the graduating class and two thousand persons, to defend this thesis by seeming attacks of the son, and, on a black-board, in the heat of the controversy, demonstrated, as I thought, as fully as any one of the problems of Euclid, the feasibility of the plan, and the truth of its being an American discovery, and Nathan Smith the author. Yet, within five years, there has been quite a controversy between two or three comparatively young men, each claiming and proving, by time (that can not lie), to be the discoverers. "There is nothing new," etc.

What brought this subject most to my mind was, in reading a "volume of forgotten lore," the article on "Fever, Malarial, Cryptogamous, Origin of; by Dr. J. K. Mitchell, of Philadelphia; Published in 1849." Whatever may be said of the truth or fallacy of the opinions given in the article, one thing is true: in certain lectures delivered before then, the opinions were asserted, and founded on certain facts and hypotheses; and had not Dr. Cowdell's Essay claimed this as a discovery, Dr. Mitchell never would have more than simply asserted, in his lectures, these facts as original. He published, and fully (as he thought) vindicated, not only the originality of the discovery, but its truth.

Dr. Cowdell is not the only claimant. Either Prof. Saulsberry, of Cleveland, or his friends here for him, claim that he is the author of this most true and most important discovery, the cryptogamous origin of fever; and many of the experiments were made here, and were observed and attested by several of our most talented physicians, very recently.

As we have remarked, the facts were set forth in lectures; and, in 1849, Dr. Mitchell issued a book, contesting the claim of Dr. Cowdell. Of course, any thing since will give way to the facts then published; and, in order to give your readers the facts, with your permission, I will copy Dr. Mitchell's remarks:

"My preference is founded on the vast number, extraordinary variety, minuteness, diffusion, and climatic peculiarity, of the fungi. The pores of these plants are not only numerous, minute, and indefinitely diffused, but they are so like to animal cells, as to have the power of penetrating into and germinating upon the most interior tissues of the human body. Introduced into the body through the stomach, or by the skin or lungs, cryptogamous poisons were shown to produce diseases of a febrile character, intermittent, remittent and continued, which were most sucessfully treated by wine and bark. As microscopic investigations become more minute, we find protophytes in disease, where, until our own time, their existence was not even suspected; as in the discharges of some kinds of dysentery, and in the *sarcini* of pyrosis. We are, therefore, entitled to believe that discovery will be, on this subject, progressive. The detection of the origin of the muscardine of the silk-worm, and a great many analogous diseases of insects, fishes and reptiles — and the demonstration of the cryptogamism of these maladies, their contagious character in one species of animals, their transfer to many other species, nay, even to vegetables themselves — all concur to render less improbable the agency of fungi in the causation of diseases of a febrile character. A curious citation was subsequently made of the fungiferous condition during epidemics and epizotics. These moulds, red, white, yellow, grey, or even black, stained garments, utensils, and pavements, made the fogs fœtid, and caused disagreeable odors and spots, even in the recesses of closets, and the interior of trunks and desks. These moulds existed even when the hygromatic state did not give to the air any unusual moisture for their sustenation and propagation. Their genus seemed to have, as many epidemics have, an inherent power of extension. The singular prevalence of malarious diseases in the autumn, is best explained by supposing them to be produced by the fungi, which grow most commonly at that season. The season of greatest protophytic activity is, in every country, the period of greatest malarious disturbance. The sickly season is in the rains of Africa, in the very dry season in Majorca and Sardinia, in the rainy season in the insular West Indies, and in the dry season of Demarara and Surinam. Even where the vegetation is peculiarly controlled, as in Egypt by the Nile, and the crytogami are thus thrown into the season of winter and spring, that season becomes, contrary to rule, the pestilential part of the year. Marshes are a safe residence by day, while they are often highly dangerous by night. In the most deadly localities of our Southern country and of Africa, the sportsman may tread the mass of a swamp by day, although at every step he extricates vast quantities of the gases, which lie entangled in mud and vegetable mould. This point, so readily explained by reference to the acknowledged nocturnal growth and power of the fungi, is a complete stumbling-block to the miasmatists. The cryptogamous theory well explains the obstruction to the progress of malaria offered by a road, a wall, a screen of trees, a veil, or a gauze curtain. It also accounts for the localization of an ague, a yellow fever, or cholera; and the want of power in steady winds to convey malarious diseases into the heart of a city, from the adjacent country. It explains, also, well, the security afforded by artificially drying the air of malarious places, the exemption of cooks and smiths from the sweating sickness, the cause of the danger from mouldy sheets, and of the sternation from old books and papers. On no other theory can we so well account, if account at all, for the phenomena of milybrand and milk-sickness, the introduction of yellow fever into northern ports, and the wonderful irregularities of the progress of cholera. The cryptogamous theory will well explain the domestications of different diseases, in different regions which have a similar climate—the plague of Egypt, the yellow fever of the Antilles, and the cholera of India. It accounts, too, for their occasional expansion into unaccustomed places, and their retreat back to their original haunts. Our hypothesis will also enable us to tell why malarious sickness is disproportionate to the character of the seasons; why it infests some tropical countries, and spares others; why the dry Maremma abounds with fevers, while the wet shores of Brazil and Australia actually luxuriate in healthfulness. The prolonged incubative period, the frequent relapses of intermittents, and the latency of the malarious poisons for months, can only be well explained by adopting the theory of a fungous causation. Finally, it explains the cause of the non-concurrence of very potent maladies, better than the chemical theory of Liebig; and shows why the earliest causes of an epidemic are commonly the most fatal." (Pp. 33–7.)

I have copied thus far, to settle priority of the claim to this theory, whether true or false; and to prove "We travel in circles."

When Vasco de Gama sailed round the Cape of Good Hope, in 1497, and when Venice lost her supremacy, and commercial prosperity dawned on Europe and England, he thought he was the first to circumnavigate Africa; but he was mistaken. He but followed Pharoah-necho, the Egyptian king, who traversed the same, two thousand years before. And now, when the world is startled at the greatness and usefulness of the canal made by the present Napoleon, connecting the Red with the Mediterranean Sea, he is only doing what was *twice* before done — and once in a greater canal than his, connecting the Nile with the Red Sea. I could multiply cases, in the discovery of brass and glass; but I only wish to prove that "There is nothing new under the sun," and that "We travel in circles;" and to demonstrate that many great discoveries are but as "Charlie's," known to all men, but really believed discoveries by their authors.

Equally as bad, as claiming other discoveries — and worse, if possible — was our Government adopting some Frenchman's great antidote for snake-bite, putting it among articles furnished to army surgeons; — a discovery of Prof. Brainard. He told me, after years of experiment with snakes in his office, and his friends abusing him roundly for their presence and fœtor, he discovered a remedy as antidotal to the poison of snakes; and, on publishing it, the Government had put up the identical prescription, under the name of *Bibron's Antidote*, and sends it even now, after he made the full explanation of the facts to the War Department. This is almost as unpleasant "as a bad picture, and worse bust." E. O. T.

---

### *Cubebic Acid.*

The active principle of *Cubebs* has been found to reside in *Cubebic acid*, a chrystallizable constituent, and not in the volatile oil or resins. From eight to thirty grains of this, in pill, in the twenty-four hours, will, it is said, cure in six days. Mild astringent injections will soon remove any remaining symptoms, in the less tractable cases.

